# Comparative Analysis of Electrophoretic Deposition and Dip Coating for Enhancing Electrical Properties of Electrospun PVDF Mats Through Carbon Nanotube Deposition

**DOI:** 10.3390/ma18163730

**Published:** 2025-08-08

**Authors:** Michał Kopacz, Piotr K. Szewczyk, Elżbieta Długoń, Urszula Stachewicz

**Affiliations:** 1 Faculty of Metals Engineering and Industrial Computer Science, AGH University of Krakow, 30-054 Krakow, Poland; mkopacz@agh.edu.pl (M.K.); pszew@agh.edu.pl (P.K.S.); 2 Faculty of Materials Science and Ceramics, AGH University of Krakow, 30-054 Krakow, Poland; dlugon@agh.edu.pl

**Keywords:** electrospun mats, EPD, dip coating, CNT, PVDF

## Abstract

Integrating carbon nanotubes (CNTs) into electrospun polyvinylidene fluoride (PVDF) fibers is a promising approach for developing conductive and multifunctional materials. This study systematically compared two CNT deposition techniques, electrophoretic deposition (EPD) and dip coating (DC), in terms of their effectiveness in modifying the surface of aligned electrospun PVDF mats. Morphological characterization revealed that EPD produced more homogeneous and compact CNT coatings. In contrast, DC resulted in discontinuous and irregular layers regardless of deposition time. A key distinction between the two methods was the tunability of the coating: EPD allowed for precise control over CNT layer thickness and mass accumulation by adjusting the deposition time. In contrast, DC showed no significant changes in thickness with longer immersion. These structural differences translated into distinct electrical behaviors. Resistance measurements showed that EPD samples exhibited a substantial decrease in resistance with increasing deposition time, from 5.9 ± 2.5 kΩ to 0.2 ± 0.1 kΩ, indicating the formation of well-connected conductive pathways. On the other hand, DC samples maintained relatively constant, higher resistance values across all conditions. Additionally, EPD-coated mats demonstrated enhanced touch sensitivity, generating higher and more stable current responses compared to DC-deposited samples. These results confirm that EPD is a more effective, tunable method for fabricating conductive CNT coatings on electrospun PVDF mats, particularly for applications in flexible electronics and wearable sensors.

## 1. Introduction

The development of multifunctional materials based on electrospun fibers opens new possibilities in areas such as smart textiles, energy harvesting, and flexible electronics [1,2,3]. These materials are valued for their large surface area, controllable porosity, and ease of surface modification [4,5]. These characteristics make them ideal for integrating specific functionalities, such as conductivity, responsiveness, and sensing capability [6]. In recent years, modifying the surfaces of electrospun fibers has become a common approach to introducing such functionalities, allowing material properties to be tailored to particular application needs [7]. One effective strategy is the deposition of carbon nanotubes (CNTs), which are known for their electrical conductivity, mechanical strength, and high aspect ratio [8]. Forming a conductive coating of CNTs on the surface of electrospun mats can transform insulating fiber networks into electroactive materials suitable for various advanced applications [9]. Polyvinylidene fluoride (PVDF) is one of the most widely used polymers for smart textiles due to its piezoelectric behavior, chemical resistance, and mechanical flexibility [10]. In addition to its functional versatility, PVDF is widely used in membrane technologies, acoustic devices, and biomedical applications due to its thermal stability, semicrystalline nature, and mechanical robustness [11]. Its molecular structure, composed of a carbon backbone with alternating fluorine and hydrogen atoms, enables the formation of distinct chain conformations (α, β, and γ) [11]. These conformations influence the crystallinity, polarity, and piezoelectric response of the material, allowing its properties to be tailored through processing conditions [12]. However, PVDF is intrinsically nonconductive, limiting its functionality in devices requiring electrical transport [13]. Coating PVDF-based mats with a layer of CNTs is a promising solution to overcome this drawback and expand their use in flexible electronics, pressure sensors, and energy storage devices [14]. Various techniques are used to deposit CNTs onto fibrous substrates [15]. Among these techniques, electrophoretic deposition (EPD) and dip coating (DC) are widely adopted due to their simplicity and scalability [16,17,18]. In EPD, an electric field moves charged CNT particles from a dispersion onto a substrate, enabling directional, uniform, and controllable deposition [19,20]. The ability to adjust layer thickness by varying the deposition time or voltage makes EPD ideal for precision applications [21]. In contrast, DC involves immersing the sample in a CNT dispersion and withdrawing it at a controlled speed [16]. Although this method is easy to implement and suitable for covering large areas, it often results in uneven or poorly adhering coatings [22], especially on porous and rough surfaces, such as electrospun mats [23]. Despite the increasing use of both methods, direct comparisons of EPD and DC for CNT deposition on electrospun fibers are limited. To address this, we performed a systematic comparison of both techniques under identical conditions, using the same CNT dispersion, substrate type, and deposition times. While DC has proven effective in other studies [14], its performance strongly depends on process-specific parameters such as withdrawal speed, surface energy, or dispersion stability. Here, our focus was to evaluate the relative effectiveness of EPD and DC under unified, reproducible conditions.

This study presents a comparative analysis of two CNT deposition methods, focusing on their impact on the morphology, thickness, and electrical performance of coatings formed on electrospun PVDF mats. The results demonstrate distinct differences in layer formation and provide practical guidelines for selecting the most effective strategy to enhance the conductivity of fibrous polymer composites.

## 2. Materials and Methods

### 2.1. Materials

Polyvinylidene fluoride (PVDF, Alfa Chemistry, Holbrook, NY, USA) with a molecular weight of 275,000 g/mol was used. A 24 wt% polymer solution was prepared using a 1:1 solvent mixture of dimethylacetamide (DMAc, Avantor, Poland) and acetone (Avantor, Poland). The solution was stirred magnetically at 47 °C for 4 h at a constant speed of 350 rpm to ensure complete dissolution. For electrophoretic deposition (EPD) and dip coating (DC), a suspension of multiwalled carbon nanotubes (MWCNTs) in isopropanol (NanoAmor, Houston, TX, USA) was used. This suspension contained 2.5% *v*/*v* MWCNTs. The MWCNTs had a stated purity above 98 wt%, outer diameters ranging from 30 to 80 nm, inner diameters of 5–15 nm, and lengths of less than 10 μm. Throughout this manuscript, the term CNTs refers specifically to these MWCNTs.

### 2.2. Electrospinning

Electrospinning was carried out using a climate-controlled EC-DIG system (IME Technologies, Waalre, the Netherlands). The PVDF solution was dispensed through a 0.8 mm inner-diameter stainless-steel needle under a constant electric field of 12 kV. The needle was positioned 14 cm from the grounded collector. The solution flow rate was set at 2 mL h^−1^. Electrospinning was conducted at 25 °C and 60% relative humidity. A foil-covered, rotating drum served as the collection surface. To achieve aligned fibers, the collector was rotated at 2500 rpm for 45 min.

### 2.3. Electrophoretic Deposition

Prior to deposition, the CNT suspension was sonicated for 30 min using an ultrasonic cleaner (Sonic-0.5, Polsonic, Warsaw, Poland) to ensure uniform dispersion. Electrospun PVDF mats (dimensions: 35 × 20 mm), attached to Al foil substrates, were used as deposition targets. Each mat was positioned centrally between two cathodes and fully submerged in the CNT suspension to a depth of 35 mm, corresponding to the total height of the mat. The total volume of CNT suspension used per deposition was 50 mL. During sample placement and removal, the mats were immersed and withdrawn from the suspension at a controlled speed of 20 mm/s to minimize disturbance of the CNT dispersion. In this configuration, the PVDF mat with aluminum foil acted as the anode, enabling deposition of CNTs on the surface. A constant voltage of 40 V was applied for 30 s, 60 s, and 90 s, corresponding to current values in the range of 0.8–1.0 mA. By applying three different deposition times, three distinct sample types were fabricated. Following the EPD process, all samples were dried under ambient conditions for 4 h and subsequently stored in an exicator.

### 2.4. Dip Coating

Before DC, the CNT suspension was sonicated for 30 min using an ultrasonic cleaner to achieve stable dispersion, following the same procedure as in the EPD method. Electrospun PVDF mats on aluminum foil substrates were then immersed in 50 mL of the CNT suspension to a depth of 35 mm. The mats were both immersed and withdrawn at a controlled speed of 20 mm/s and held in the suspension for one of three durations: 30, 60, or 90 s. The same parameters were used as in the EPD process to ensure direct comparability. After withdrawal, the samples were left to dry under ambient conditions for 4 h and subsequently stored in a desiccator. As with EPD, three distinct sample types were obtained based on coating time.

### 2.5. Scanning Electron Microscopy and Mass Measurement

Surface and cross-sectional morphology of the electrospun PVDF fibers was characterized using a field-emission scanning electron microscope (SEM, Merlin Gemini II, Zeiss, Jena, Germany) equipped with a secondary electron (SE) detector. For cross-sectional imaging, samples were immersed in liquid nitrogen and then fractured using a surgical scalpel to preserve the internal structure of the mats. Both surface and cross-section samples were subsequently sputter-coated with an 8 nm gold layer using a rotary-pumped coater (Q150RS, Quorum Technologies, Lewes, UK) to improve conductivity. SEM imaging was conducted at an accelerating voltage of 3 kV, a beam current of 150 pA, and a working distance between 2 and 5 mm. Fiber diameter distributions and corresponding histograms were analyzed using OriginPro software (version 2025, OriginLab, Northampton, MA, USA). A normal distribution curve was fitted to the data to evaluate the statistical consistency of fiber diameters.

To evaluate material accumulation during CNT deposition, five samples were prepared for each EDP and DC method at three deposition times of 30, 60, and 90 s. The initial mass of each electrospun PVDF mat was measured before coating. Then, the samples were subjected to CNT deposition using either EPD or DC under the selected time conditions. After coating, the mats were left to dry for 4 h under ambient conditions and then stored in an exicator to remove residual moisture. After drying, the samples were reweighed, and the mass difference was calculated for each sample.

### 2.6. Electrical Resistance Measurements

The resistance of CNT-coated PVDF samples obtained via EPD and DC was evaluated using the two-point probe method. Measurements were carried out with a high-precision digital multimeter (DMM6500, Keithley Instruments, Solon, OH, USA) connected to metallic electrodes. Resistance values were recorded for two electrode spacings: 1 and 2 cm. All measurements were performed under ambient conditions. In addition, through-thickness resistance was measured for representative EPD60 and DC60 samples to assess vertical charge transport across the mat. The measurements were performed using an electrometer (Keithley 6517B, Solon, OH, USA) integrated into a mechanical setup with a linear motor (LinMot—P04, Lake Geneva, WI, USA). Each sample was placed between two flat metallic electrodes and pressed with a constant force of 10 N to ensure consistent contact. For each sample, 100 resistance measurements were recorded. The mean resistance and standard deviation were calculated using OriginPro software (2023, OriginLab, Northampton, MA, USA) to ensure statistical reliability.

### 2.7. Sensor Response Analysis

To evaluate the touch-sensing capabilities of the CNT-coated electrospun PVDF mats, two complementary experiments were performed to assess both piezoresistive and triboelectric responses. First, a piezoresistive response test was conducted using manual finger tapping to simulate real-world tactile stimuli. The measurement was carried out on three types of samples: EPD60, DC60, and pristine electrospun PVDF. Each sample (12 × 15 mm) was placed between two metallic electrodes spaced 15 mm apart and connected to a digital multimeter (Keithley 6500, Solon, OH, USA) configured for two-point resistance measurements. Real-time resistance changes were recorded during tapping to assess the materials’ responsiveness to mechanical input. The measurement setup is illustrated in Figure S1, and a real-time demonstration is provided in the updated Movie S1 in the Supplementary Materials. This configuration ensures direct comparison between CNT-coated and uncoated mats, highlighting the essential role of the CNT layer in enabling touch sensitivity via piezoresistive mechanisms. Second, a voltage response test was performed to evaluate the triboelectric behavior of the same samples using a linear motor-based tapping setup (LinMot—P04, Lake Geneva, WI, USA), applying a consistent contact force of 20 N at a frequency of 1.5 Hz. In this setup, the sample was connected to the HI terminal of a high-impedance electrometer (Keithley 6517B, Solon, OH, USA) via an adhesive metallic electrode on the backside. A nylon 6 fabric, chosen for its high triboelectric contrast with PVDF, was used as the counter-electrode and mounted on a metal plate connected to the LO terminal. Open-circuit voltage (Voc) signals generated by the contact–separation process were recorded and analyzed to compare the output characteristics of pristine and CNT-modified samples.

## 3. Results and Discussion

**Figure 1:**
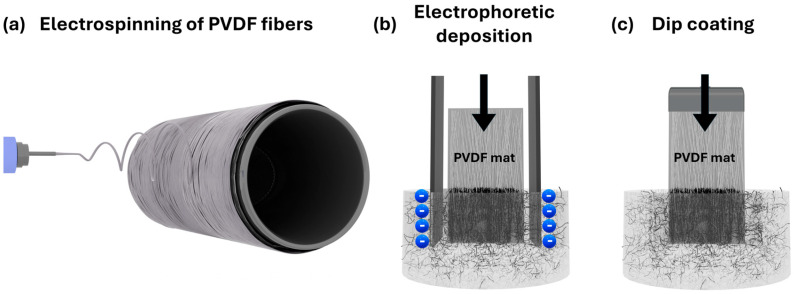
Schematic illustration of the processing steps used in this study: (**a**) electrospinning of PVDF mats, (**b**) electrophoretic deposition of CNTs onto electrospun PVDF mats (blue spheres represent schematic illustration of electric charges), and (**c**) dip coating of CNTs onto electrospun PVDF mats.

The fabrication of conductive electrospun mats was carried out using a two-step process, schematically illustrated in Figure 1. First (Figure 1a), PVDF fibers were produced via electrospinning to create porous, aligned fibrous mats. In the second step, a conductive CNT layer was deposited onto the fiber surface using either EPD (Figure 1b) or DC (Figure 1c). Both techniques were applied in order to directly compare their effectiveness in forming homogeneous, conductive CNT coatings on electrospun substrates. The following sections present a detailed analysis of the resulting morphology, layer formation, and electrical performance.

### 3.1. Morphology Characterization

**Figure 2:**
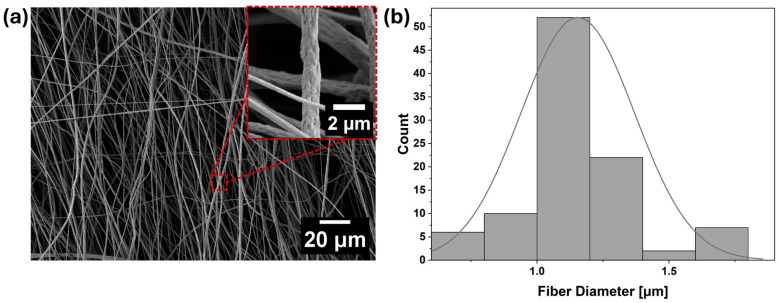
(**a**) SEM micrograph of aligned PVDF electrospun fibers. (**b**) Histogram representing the fiber diameter distribution of the electrospun PVDF mat.

Aligned electrospun PVDF fibers were fabricated as the base material for further surface modification via CNT deposition. The use of aligned fibers was motivated by their improved mechanical performance and enhanced electrical properties because fiber alignment enables more continuous and efficient conductive pathways for directional charge transport within the fibrous network [24]. The electrospun PVDF fibers exhibited a slightly wrinkled surface morphology, as shown in Figure 2a. This feature is typically associated with phase separation and vapor-induced instabilities that occur during fiber solidification under moderate humidity conditions [25]. In the present study, electrospinning was carried out at 60% relative humidity and 25 °C, conditions that are known to promote such effects. A previous study reported similar morphological characteristics, where elevated humidity levels led to wrinkling and porosity formation in PVDF fibers due to vapor-phase interactions during jet elongation and solvent evaporation [26]. Quantitative analysis of the fiber diameters revealed an average value of 1.12 ± 0.21 µm, consistent with values reported in previous studies [27]. Figure 2b shows the corresponding diameter distribution as a histogram, demonstrating a narrow and symmetric spread.

**Figure 3:**
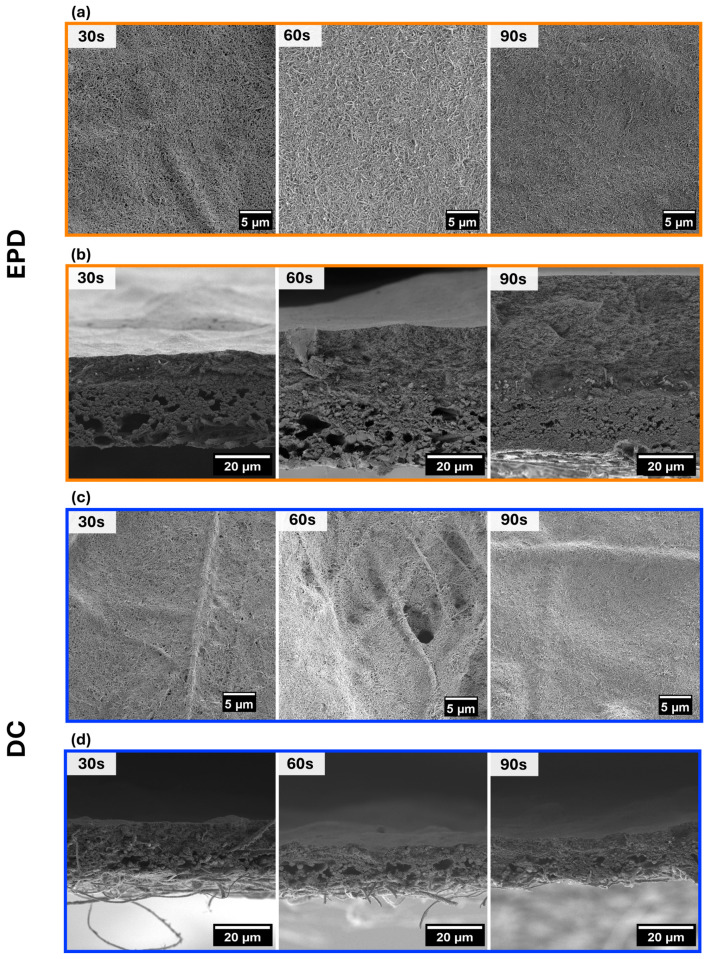
SEM micrographs of the CNT layers deposited on electrospun PVDF fibers: (**a**) top surface view of the samples after the EPD technique, (**b**) top surface view of the samples after the DC method, (**c**) cross-section of the samples after the EPD technique, and (**d**) cross-section of the samples after the DC method. Orange and blue frames indicate EPD- and DC-modified samples, respectively.

PVDF mats coated with CNTs using EPD and DC are referred to as EPDX and DCX, respectively, where X indicates the deposition time of 30, 60, or 90 s. Figure 3 shows SEM images of the morphology of CNT coatings formed on electrospun PVDF mats using EPD or DC. There is a clear difference in layer uniformity between these two techniques. In EPD60 and EPD90 (Figure 3a), the CNTs form a uniform, continuous film that completely covers the fiber surface. The deposited layer appears dense and well-integrated with the fibrous structure, indicating strong adhesion and effective particle accumulation during the EPD process [28]. However, for EPD30, localized thickening of the CNT layer is observed around individual fibers. As the deposition time of CNTs increases, these accumulations transition into a more homogeneous CNT layer that covers the entire fiber surface and minimizes exposed regions, see Figure 3a for EPD60 and EPD90.

In contrast, the DCX samples display significantly less uniformity. As seen in Figure 3c, many areas of the PVDF mat remain uncovered, with individual fibers exposed through a thin and fragmented CNT layer. The CNTs tend to aggregate irregularly, forming a patchy, discontinuous coating. Increasing the deposition time from DC30 to DC90 does not visibly improve the homogeneity of the CNT layer. Even in the DC90 sample, the PVDF fibers are clearly visible under a thin, uneven CNT film, see Figure 3c. This indicates that dip coating offers limited control over layer formation and does not support time-dependent growth in the same way that EPD does.

**Figure 4:**
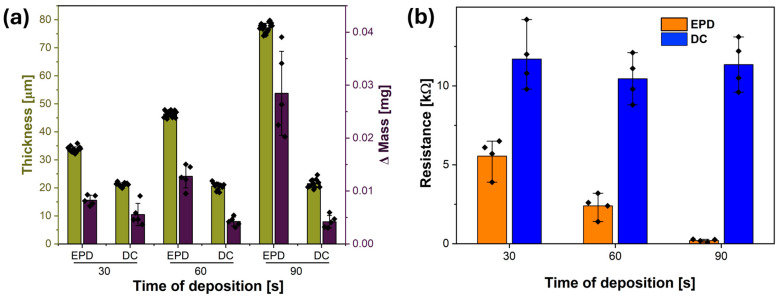
Results of EPDX and DCX samples: (**a**) Thickness and mass difference measurements of the CNT layers deposited by EPD and DC on PVDF electrospun mats. (**b**) Resistance measurements obtained with a 1 cm electrode spacing. Bars represent mean values, with individual data points shown as rhomboid symbols overlaid on each bar.

The cross-sectional SEM images presented in Figure 3 further illustrate the differences in CNT layer formation between the EPD and DC methods. As shown in Figure 3b, samples from the EPD series exhibit a clear and progressive increase in CNT layer thickness with deposition time. In EPD30, the CNT film is already visible as a compact layer adhering to the surface of the PVDF mat, with slight variations in thickness corresponding to the fiber architecture. For EPD60, the layer becomes more continuous and thicker, while EPD90 reveals a well-developed, homogeneous CNT coating with significantly increased thickness across the entire sample. The results in Figure 4a quantitatively confirm this trend, showing that CNT layer thickness increases from 28.8 ± 4.3 µm in EPD30 to 74.5 ± 8.6 µm in EPD90. These results demonstrate that EPD enables precise control over CNT film growth by adjusting deposition time, allowing for tunable surface functionalization based on application requirements. Furthermore, the time-dependent growth is confirmed by the increase in mass of the samples: the mass gain rises from 8.2 ± 0.9 mg for EPD30 to 28.4 ± 6.1 mg for EPD90, indicating continuous CNT deposition over time, see Figure 4a.

This time-dependent film growth is further supported by mass measurements. The average mass gain increases from 8.2 ± 0.9 mg for EPD30 to 28.4 ± 6.1 mg for EPD90, indicating continuous and efficient CNT accumulation. Together, these results confirm that EPD enables precise, tunable CNT deposition by adjusting deposition time alone, making it a suitable surface functionalization approach in application-specific contexts.

By comparison, the cross-sectional images in Figure 3d show that the samples obtained via DC exhibit minimal changes in CNT layer thickness regardless of immersion time. The deposited CNT film remains thin, irregularly distributed, and structurally inconsistent across the mat. The data shown in Figure 4a support this observation, showing that the CNT layer thickness in dip-coated samples remains nearly constant, ranging from 21.1 ± 1.1 µm in DC30 to 21.5 ± 1.5 µm in DC90. This minimal variation indicates that prolonged immersion time does not result in additional material buildup, as the dip-coating mechanism is governed primarily by the number of dipping cycles rather than the duration of a single immersion [29]. A similar trend is evident in the mass change measurements, which slightly decrease from 5.5 ± 1.8 mg for the DC30 mat to 4.2 ± 1.0 mg for the DC90 mat. While the reduction may fall within experimental uncertainty, the overall lack of increase confirms the limited capability of dip coating to produce thicker or more uniform CNT layers on porous electrospun PVDF mats [19,30]. The results clearly demonstrate a fundamental difference between these two deposition techniques. EPD enables time-dependent control over CNT layer thickness and material accumulation, allowing for gradual, uniform coating buildup.

### 3.2. Electrical Conductivity Results

The electrical behavior of EPD and DC mats was examined using the two-point probe method to investigate the influence of CNT layer morphology on the resulting electrical conductivity. Resistance values were collected for samples prepared via EPD and DC, using electrode spacings of 1 cm. The results are visualized in Figure 4b.

The results revealed a clear correlation between the deposition method, time, and the resulting electrical behavior of the samples. For EPD-coated mats (EPD30, EPD60, and EPD90), substantial decreases in resistance were observed as deposition time increased. At an electrode spacing of 1 cm, resistance decreased from 5.9 ± 2.5 kΩ for EPD30 to 0.2 ± 0.1 kΩ for EPD90, which is more than a one-order-of-magnitude reduction. An increasing concentration of CNTs deposited on the fiber surface corresponds to a decline in resistance, resulting in improved electrical conductivity [31]. This strong time-dependent trend suggests the progressive formation of a denser, more interconnected CNT layer that enhances charge transport pathways across the fibrous structure [8]. Dip-coated samples (DC30, DC60, and DC90) exhibited consistently high resistance values that remained relatively constant regardless of immersion time. For a spacing of 1 cm, resistance ranged from 10.5 ± 2.2 kΩ to 12.3 ± 2.5 kΩ. This indicates that longer immersion during dip coating does not lead to improved CNT coverage or connectivity [32]. The consistently high resistance suggests that the CNT films deposited via DC are too sparse and discontinuous to form an effective conductive layer on the PVDF mat [33,34].

In addition to surface resistance measurements, through-thickness resistance was also evaluated for the EPD60 and DC60 samples to further compare their electrical properties. The EPD60 sample exhibited a significantly lower resistance of 10.70 ± 0.01 Ω, while the DC60 sample showed a more than fourfold higher resistance of 46.68 ± 0.03 Ω. This finding further confirms that EPD enables more uniform CNT penetration and better connectivity throughout the fibrous mat, whereas the DC method results in a less integrated, surface-limited coating.

These electrical measurements strongly correlate with the earlier discussed morphological and structural observations. As shown in Figure 3, CNT layers produced by EPD become increasingly homogeneous and thick over time, whereas layers formed via DC remain thin and discontinuous. The data suggests that EPD not only facilitates the formation of uniform coatings but also enables precise control over electrical functionality by adjusting deposition time. In contrast, the DC method provides limited electrical tunability, likely due to poor film formation on the electrospun PVDF mats.

To contextualize the performance of our EPD-modified mats, we compare our results with previously reported PVDF/CNT composites fabricated using conventional film-forming techniques. For example, a solution-cast PVDF film containing 4 wt% unfunctionalized CNTs (4ufCNT-S) exhibited a resistance of 248 Ω, while a melt-mixed film with 4 wt% functionalized CNTs (4fCNT-M) showed a resistance of 305 Ω [35]. Another notable system is a sandwich-structured strain sensor composed of electrospun PDMS/PVDF and silver nanowires (PDMS/PVDF-AgNWs), which reached an initial resistance of 281 Ω at 40 wt% AgNW [36]. In comparison, the EPD90 sample developed in this work achieved a lower resistance of 0.2 ± 0.1 kΩ, despite being based on a highly porous and flexible electrospun structure. These results demonstrate that the EPD method enables the formation of conductive networks that are not only competitive with, but in some cases outperform, dense or hybrid film architectures, while maintaining high flexibility and breathability.

### 3.3. Sensor Sensitivity Test

**Figure 5:**
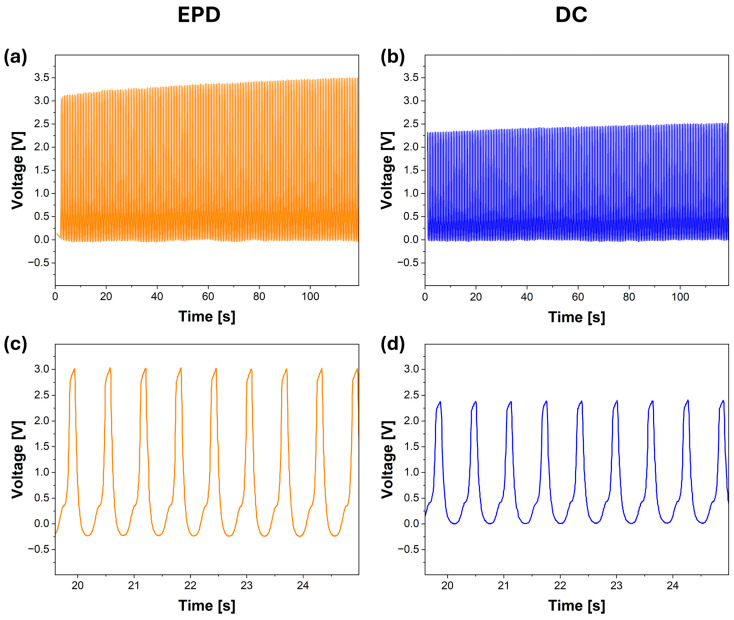
Voltage response of tapping sensors based on electrospun aligned PVDF fibers coated with CNTs: (**a**) voltage signal over a 180 s period for sample EPD60, (**b**) voltage signal over a 180 s period for sample DC60, (**c**) zoomed-in response over a 5 s window for EPD60, (**d**) zoomed-in response over a 5 s window for DC60. Orange and blue indicate EPD- and DC-modified samples, respectively.

To investigate how the CNT deposition method influences the touch-sensing capability of the mats, EPD60 and DC60 samples were tested using a linear motor-based tapping system. As shown in Figure 5a and b, both samples exhibited a stable and repeatable voltage response over the entire 180 s measurement period, confirming their functionality as touch sensors. A closer examination of the voltage variation during individual tapping events revealed notable differences in signal amplitude. The EPD60 sample (Figure 5c) generated sharp and consistent voltage peaks reaching more than 10 V, whereas the DC60 sample (Figure 5d) produced lower amplitude signals, with peak values reaching around 9 V. However, the consistently lower voltage values observed for the DC60 sample indicate reduced sensitivity, likely due to its thinner and less uniform CNT layer deposited on the PVDF mat.

For reference, a pristine electrospun PVDF mat deposited on aluminum foil was subjected to the same tapping protocol. The resulting voltage signal (see Figure S2 in the Supplementary Materials) reached up to 20 V, significantly higher than that observed for CNT-coated mats. This elevated output is attributed to the triboelectric effect, in which contact between the dielectric PVDF surface and the nylon 6 counter-surface leads to the accumulation of electrostatic charges [10]. In the absence of a conductive network, these charges remain localized on the PVDF fiber surface, resulting in a pronounced open-circuit voltage detected by the electrometer [37].

In contrast, the CNT-coated samples (EPD60 and DC60) displayed markedly reduced voltage outputs due to the formation of continuous conductive pathways across the fiber surface [38]. The deposited CNT layer lowers the surface impedance and facilitates the rapid dissipation of triboelectric charges, thereby preventing significant potential buildup [39]. Notably, while the pristine PVDF mat requires an external electrode to function in the triboelectric mode, the conductive CNT-coated mats serve as self-electrodes, enabling touch sensing without the need for additional charge collection layers [40]. Although the CNT-coated mats can still operate in triboelectric mode, we subsequently evaluated their performance as piezoresistive sensors, where pressure-induced resistance changes within the CNT network offer a more robust and practical sensing mechanism [40]. This conclusion is further supported by the results presented in Movie S1 in the Supplementary Materials, where the real-time resistance response of all three samples, EPD60, DC60, and pristine PVDF, is shown under gentle finger tapping. Both EPD60 and DC60 samples exhibit distinct, measurable changes in resistance during tapping, confirming their piezoresistive behavior. In contrast, the pristine PVDF mat shows no detectable signal, consistent with its inherently high electrical resistance and lack of conductive pathways. These findings emphasize that the introduction of a CNT network is essential for enabling touch-sensing functionality in the mats and confirm that piezoresistivity enables reliable detection of low-pressure mechanical stimuli such as human touch in the CNT-modified samples. Additionally, Figure S3 in the Supplementary Materials presents a schematic of the electrical setup used for triboelectric measurements on EPD60, DC60, and pristine PVDF samples. The experiment follows the standard contact–separation mode used in triboelectric nanogenerators. Mechanical tapping between the sample and a nylon 6 surface induces charge transfer, recorded as a Voc.

## 4. Conclusions

In this study, the effectiveness of two CNT deposition techniques, EPD and DC, was evaluated for forming conductive layers on electrospun PVDF mats. While both methods enabled CNT integration onto the fibrous surface, EPD produced significantly more homogeneous, compact, and continuous coatings compared to the thin and nonuniform layers obtained via DC. A key advantage of EPD was the ability to control layer thickness through deposition time, with values increasing from 28.8 ± 4.3 µm for the EPD30 mat to 74.5 ± 8.6 µm for the EPD90 mat, where DC showed minimal variation in thickness regardless of immersion duration. These morphological differences were directly reflected in the electrical performance. CNT-coated PVDF fibers made using EPD exhibited a substantial, time-dependent decrease in resistance from 5.9 ± 2.5 kΩ for EPD30 to 0.2 ± 0.1 kΩ for EPD90, indicating efficient formation of a conductive CNT network. In contrast, DC samples maintained higher and relatively stable resistance values (10.5 ± 2.2 kΩ to 12.3 ± 2.5 kΩ), highlighting the method’s limited ability to form percolated structures. Notably, EPD60 also exhibited a markedly lower through-thickness resistance (10.70 ± 0.01 Ω) compared to DC60 (46.70 ± 0.03 Ω), confirming enhanced CNT connectivity across the mat. This improved conductivity translated into superior sensing characteristics: EPD60 generated higher voltage outputs (~9 V) under repeated tapping than DC60 (~10 V). Overall, the results demonstrate that EPD is a more robust and tunable method for creating conductive CNT layers on electrospun PVDF mats and is better suited for applications requiring reliable electrical performance, including wearable platforms.

## Data Availability

The original contributions presented in this study are included in the article/Supplementary Material. Further inquiries can be directed to the corresponding author.

## Supplementary Materials

The following supporting information can be downloaded at: https://www.mdpi.com/article/10.3390/ma18163730/s1, Figure S1: Photo of the touch-sensing setup used for manual finger tapping tests of the electrospun PVDF samples coated with CNTs; Figure S2: Voltage response of tapping sensors based on electrospun aligned PVDF fibers: (a) voltage signal over a 180 s period for the sample, (b) zoomed-in response over a 5 s window for the sample; Figure S3: Schematic illustration of charge flow during triboelectric tapping measurement in our samples; Movie S1: The sensing application of the PVDF mat and PVDF mat coated with CNTs.
