# Supplementary material for: Comparative Analysis of Electrophoretic Deposition and Dip Coating for Enhancing Electrical Properties of Electrospun PVDF Mats Through Carbon Nanotube Deposition
Source: Materials (Basel). 2025 Aug 8;18(16):3730. doi: 10.3390/ma18163730 (PMC12387443; doi:10.3390/ma18163730)
Supplement: Supplementary file 1 [file materials-18-03730-s001.zip › materials-3774069_Supplementary Materials-EPD-vs-DC-20250802_2.pdf]

Supplementary Materials to

Comparative Analysis of Electrophoretic Deposition and Dip Coating for enhancing Electrical Properties of Electrospun PVDF Mats through Carbon Nanotube Deposition

Michał Kopacz [1], Piotr K. Szewczyk [1], Elżbieta Długoń [2] and Urszula Stachewicz [1*]

[1]*Faculty of Metals Engineering and Industrial Computer Science, AGH University of Krakow, Kraków, 30-054, Poland*

[2]*Faculty of Materials Science and Ceramics, AGH University of Krakow, Kraków, 30-054, Poland*

*E-mail: ustachew@agh.edu.pl

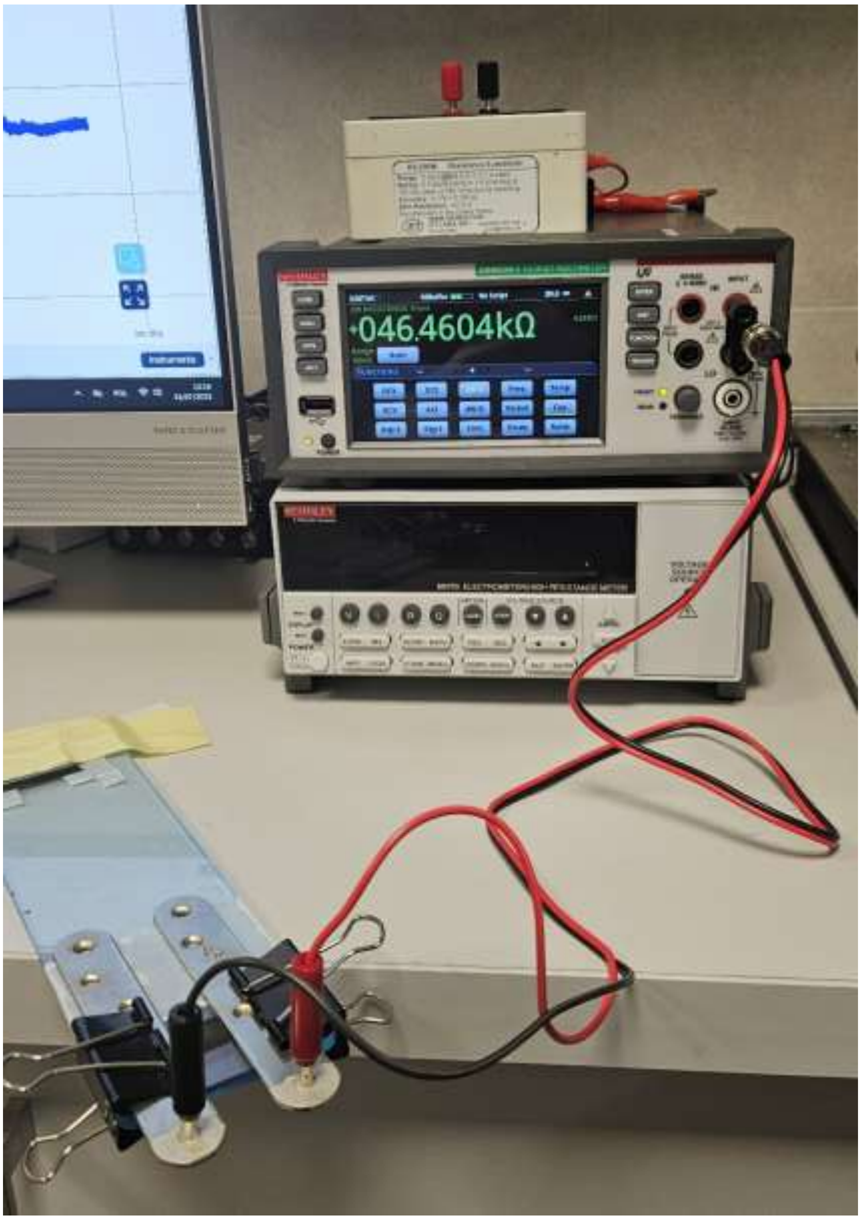

Figure S1. Photo of the touch-sensing setup used for manual finger tapping tests of the electrospun PVDF samples coated with CNTs.

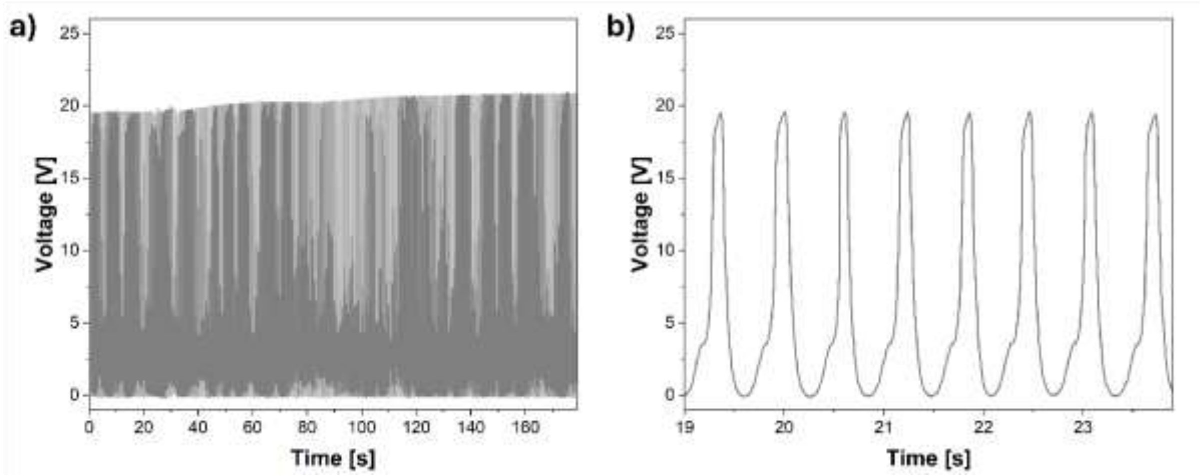

Figure S2. Voltage response of tapping sensors based on electrospun aligned PVDF fibers **a)** voltage signal over a 180 s period for the sample, **b)** zoomed-in response over a 5 s window for the sample.

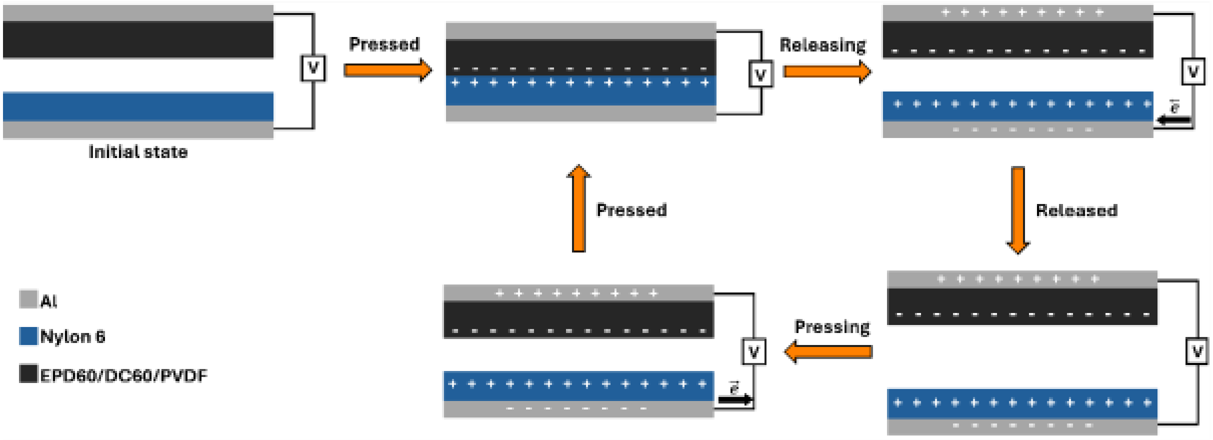

Figure S3. Schematic illustration of charge flow during triboelectric tapping measurement in our samples.
